# Role of Stearoyl-CoA Desaturase 1 in Cardiovascular Physiology

**DOI:** 10.3390/ijms24065531

**Published:** 2023-03-14

**Authors:** Volodymyr V. Balatskyi, Pawel Dobrzyn

**Affiliations:** Laboratory of Molecular Medical Biochemistry, Nencki Institute of Experimental Biology of Polish Academy of Sciences, 02-093 Warsaw, Poland; v.balatskyi@nencki.edu.pl

**Keywords:** monounsaturated fatty acids, cardiac lipid metabolism, lipogenesis, substrate utilization

## Abstract

Stearoyl-CoA desaturase is a rate-limiting enzyme in the synthesis of monounsaturated fatty acids. Monounsaturated fatty acids limit the toxicity of exogenous saturated fats. Studies have shown that stearoyl-CoA desaturase 1 is involved in the remodeling of cardiac metabolism. The loss of stearoyl-CoA desaturase 1 reduces fatty acid oxidation and increases glucose oxidation in the heart. Such a change is protective under conditions of a high-fat diet, which reduces reactive oxygen species-generating β-oxidation. In contrast, stearoyl-CoA desaturase 1 deficiency predisposes individuals to atherosclerosis under conditions of hyperlipidemia but protects against apnea-induced atherosclerosis. Stearoyl-CoA desaturase 1 deficiency also impairs angiogenesis after myocardial infarction. Clinical data show a positive correlation between blood stearoyl-CoA Δ-9 desaturation rates and cardiovascular disease and mortality. Moreover, stearoyl-CoA desaturase inhibition is considered an attractive intervention in some obesity-associated pathologies, and the importance of stearoyl-CoA desaturase in the cardiovascular system might be a limitation for developing such therapy. This review discusses the role of stearoyl-CoA desaturase 1 in the regulation of cardiovascular homeostasis and the development of heart disease and presents markers of systemic stearoyl-CoA desaturase activity and their predictive potential in the diagnosis of cardiovascular disorders.

## 1. Introduction

Lipid metabolism is essential for maintaining heart function. Fatty acids (FAs) are the primary source of energy for the healthy adult heart. Even under pathological conditions, when FA utilization is reduced, the utilization of other energy substrates does not exceed the level of FA β-oxidation [1,2]. The heart utilizes exogenous FAs that are actively taken up by specialized transporters or endogenous FAs that derive from the lipolysis of triacylglycerols (TAGs) or diacylglycerols (DAGs) [3,4]. Fatty acids are not only substrates for mitochondrial adenosine triphosphate (ATP) production but also ligands for peroxisome proliferator-activated receptor (PPAR) transcription factors that maintain the heart’s oxidative metabolic pattern. Interestingly, monounsaturated FAs (MUFAs) and polyunsaturated FAs (PUFAs) are PPAR activators, whereas saturated FAs (SFAs) are PPAR inhibitors [5]. Impairments in cardiac lipid metabolism are often observed in various pathological conditions or may be primary pathogenetic factors [1,2].

Despite the high rate of FA utilization, the heart has a limited capacity to synthesize FAs de novo. The de novo synthesis of FAs in the heart appears to have regulatory rather than metabolic functions. The ablation of FA synthase (FAS), the enzyme that synthesizes palmitic acid, leads to intolerance of the heart to pressure overload and age-dependent stress. Importantly, exogenous FAs cannot rescue cardiac function in *Fas* knockout mice [6]. Another de novo lipogenesis enzyme is Δ9-desaturase or stearoyl-CoA desaturase (SCD), which introduces a double bond mainly in palmitic and stearic acids to form palmitoleic and oleic acids, respectively [7]. SCD is a four-span transmembrane protein that is located in the endoplasmic reticulum (ER). A characteristic feature of SCD are three histidine-containing motifs that form two zinc clusters that are required for catalysis [8,9]. NADP-cytochrome b5 reductase and cytochrome b5 are required for SCD-mediated catalysis and electron flow. Molecular oxygen is the terminal electron acceptor and is reduced to H_2_O [7]. The mouse genome contains four *SCD* genes: *Scd1*, *Scd2*, *Scd3*, and *Scd4*. The human genome has two *SCD* genes: *SCD1* and primate-specific *SCD5* [10,11,12]. SCD1, SCD2, SCD4, and SCD5 utilize stearoyl-CoA as the preferred substrate over palmitoyl-CoA, whereas SCD3 preferentially desaturates myristoyl-CoA and palmitoyl-CoA [10,11]. SCD isoforms have different developmental and tissue expression and are differentially regulated by extracellular signals [10,12,13,14,15].

SCD1 regulates body metabolism, and its loss has been shown to have anti-obesity effects. Mice with global *Scd1* deletion or that are treated with anti-*Scd1* oligonucleotides are resistant to high-fat diet (HFD)-induced obesity and do not develop insulin resistance. They have a hypermetabolic phenotype, with higher energy expenditure and a higher rate of β-oxidation in liver, skeletal muscle, and adipose tissue [16,17,18]. The downregulation of SCD1 expression mediates metabolic effects of leptin [19]. Additionally, SCD1 ablation promotes beige adipose tissue formation [20]. Moreover, the anti-obesity effects of global *Scd1* deletion may partially derive from impairments in skin integrity [21,22]. Mice with a global *Scd1* knockout develop subclinical hyperthyroidism, which may be partly responsible for the obesity-resistant phenotype [23]. Additionally, SCD1 maintains normal blood homeostasis through hepatic gluconeogenesis and the production of very-low-density lipoprotein and low-density lipoprotein [24,25]. The muscle-specific overexpression of *Scd1* enhances exercise capacity, likely through an increase in mitochondrial biogenesis and FA oxidation [26]. SCD1 also regulates basic physicochemical properties of cell membranes and several intercellular signaling pathways and processes. SCD-derived MUFA incorporation into phospholipids increases membrane fluidity, and its activity opposes SFA-induced membrane rigidification [26,27]. SCD1 is involved in autophagy regulation through MUFA production and maintains the fusion of autophagosomes with lysosomes [28]. The palmitoleic acid that is produced by SCD is required for the lipidation and activation of Wingless-related integration site (WNT) ligands [29]. In brown adipose tissue, skeletal muscle, and the heart, SCD1 deficiency increases the insulin sensitivity of these tissues through the activation of insulin receptor signaling [30,31,32]. Moreover, SCD1 in these tissues regulates two energy-sensing pathways: adenosine monophosphate-activated protein kinase (AMPK)/Sirt1 and PPAR [32,33].

The present review focuses on the role of SCD1 in cardiovascular system function. We discuss the SCD-dependent reprogramming of pathways of cardiac metabolism and the involvement of SCD in maintaining vascular homeostasis. We also review clinical data on the link between markers of systemic SCD activity and cardiovascular pathophysiology and the predictive diagnostic potential of plasma markers of SCD activity.

## 2. SCD1 as a Regulator of Cardiac Function and Metabolism

The heart is an omnivorous organ using all substrates: glucose, FA, ketone bodies, and amino acids. The wide range of energy substrates and the precise regulation of the heart’s metabolism allow it to respond to various stimuli and provide adequate blood circulation [1,2]. Impaired cardiac metabolism itself can cause cardiac pathology or affect the development of heart disease caused by other factors. In this chapter, we will focus on the role of SCD1 in the pathogenesis of heart disease, the role of SCD1 in regulating cardiac metabolism, and the manipulation of SCD activity as a promising therapeutic intervention in lipotoxic cardiomyopathy.

### 2.1. Expression and Regulation of SCD Isoforms in the Heart

Three isoforms of SCD are expressed in the mouse heart: *Scd1*, *Scd2*, and the heart-specific *Scd4*. Only one isoform, *SCD1*, is expressed in the human heart [10]. All three isoforms have the same substrate specificity, but Scd4 has the lowest activity among all isoforms that are expressed in the heart [11]. Moreover, *Scd4* expression is differentially regulated compared with the other isoforms. Like *Scd1* and *Scd2*, *Scd4* is induced by a carbohydrate diet and liver X receptor α (LXRα) agonists but is not suppressed by PUFA [10]. LXRα induces Scd1 expression in a Sirt1-dependent manner in response to SFA [34]. Glucose, palmitic acid, stearic acid, and insulin also stimulate *Scd1* expression in cardiomyocytes [35,36]. Peripheral blood-born leptin suppresses only *Scd4* expression, and this appears to be a direct action of leptin on cardiomyocytes via a signaling cascade that is activated upon leptin binding to receptors. Subcutaneous leptin administration decreases cardiac *Scd4* expression in leptin-deficient mice [10]. In contrast, the activation of the leptin signaling pathway in the central nervous system via intracerebroventricular administration inhibits cardiac de novo FA synthesis. This leads to neuronal activation of the cardiac PPARβ/δ signaling pathway and suppresses the expression of genes that are involved in FA synthesis, particularly *Scd1* [37].

### 2.2. SCD1 Involvement in the Development of Cardiac Pathologies

The impairment of SCD activity contributes to the development of heart failure because its activity is often impaired in patients with heart pathologies. SCD1 is elevated in the heart in patients with diabetes and heart failure [35,38]. *Scd1* mRNA levels are unchanged or reduced in hypertrophied hearts but are elevated at the onset of heart failure in various mouse models [38,39,40,41]. *Scd1* expression also increases in the rat heart after a high-sucrose diet but without the onset of cardiac symptoms [35]. The progression of cardiac dysfunction in spontaneously hypertensive rats (SHRs) is associated with the inhibition of de novo FA synthesis. Moreover, SCD1 levels and activity decrease, leading to higher SFA content in cardiac cellular lipids [41]. Interestingly, SHRs exhibit diurnal changes in blood pressure and heart rate. These abnormalities are associated with impairments in the cyclic light/dark expression of cardiac metabolic regulators, namely PPARα and PPARγ. Interestingly, in control rats, *Scd1* expression does not show diurnal cyclicity, whereas in SHRs, *Scd1* mRNA levels are unaffected in the light phase but increase in the active dark phase [42]. Rats with hereditary hypertriglyceridemia exhibit impairments in FA metabolism in the heart. In particular, they exhibit high cardiac levels of TAGs, DAGs, cholesteryl esters, and lysophosphatidylcholine. SCD expression and activity also increase, which is reflected by the higher desaturation of Δ-9 phospholipids and cholesteryl esters [43]. Cardiac dysfunction in β-carotene-15,15′-dioxygenase-deficient mice that is caused by the inhibition of the retinoic acid signaling pathway is associated with lower TAGs levels and the activation of FA synthesis de novo, with a two-fold increase in *Scd1* mRNA levels [44]. The mismatch between FAs and TAGs synthesis leads to the accumulation of cardiotoxic ceramides and can cause heart disease. Heterozygosity for CTP:phosphoethanolamine cytidylyltransferase, a key enzyme for de novo phosphatidylethanolamine synthesis, causes male-specific cardiac hypertrophy and hypertension. An increase in the remodeling of phospholipids with an increase in the content of n-6 PUFA in males is a major pathogenic factor that causes gender-dependent cardiac hypertrophy, but only females exhibit an almost two-fold increase in SCD1 protein levels [45]. An increase in SCD1 content may be one of the factors that counteract phospholipid remodeling and prevent heart hypertrophy in females. Maternal pre-pregnancy obesity predisposes to early cardiovascular disease in offspring [46] and increases SCD1 levels in offspring hearts [47]. Additionally, higher rates of TAGs synthesis in the heart that are attributable to the upregulation of diacylglycerol acyltransferase 1 (DGAT1) are associated with an increase in the expression of *Scd1* and *Scd2* but not *Scd4* [48]. The knockout of *tissue inhibitor of metalloproteinase 3* (*Trim3*) in atherosclerosis-prone apolipoprotein E (ApoE)-deficient mice leads to cardiac dysfunction and worsens cardiac remodeling after myocardial infraction. Double mutant mice develop cardiac steatosis and impairments in FA oxidation on a chow diet, which is exacerbated by a HFD. These mice also exhibit a decrease in PPARα and *Scd1* mRNA levels [49]. Both *PPARα* and *Scd1* ablation are associated with a decrease in β-oxidation in the heart [32,50] and might contribute to impairments in cardiac function in *Trim3* knockout mice. These data indicate the involvement of SCD activity in the progression of cardiac dysfunction. However, the downregulation or upregulation of SCD expression depends on the exact nature of cardiac dysfunction, and the consequences of such changes should be clarified in future studies.

De novo FA synthesis is also involved in physiological cardiac hypertrophy development in response to exercise. Endurance training leads to the activation of the Akt/sterol regulatory element-binding protein-1c (SREBP-1c) axis, stimulating the expression of lipogenic genes (*Fas* and *Scd1*). An increase in de novo FA synthesis appears to play a regulatory role in physiological cardiac remodeling, in which lipogenesis was not impaired in pathologically hypertrophied hearts [40]. Moreover, de novo FA synthesis is required for the proliferation of neonatal atrial cardiomyocytes. Hyperoxia inhibits cardiomyocyte proliferation, which is associated with a reduction in the expression of genes that are involved in FA synthesis, particularly *Fas* and *Scd1*, in an SREBP-1-independent manner. The overexpression of *Scd1* and *Fas* is sufficient to rescue HL1 cardiomyocyte proliferation under hypertoxic conditions [51]. De novo FA synthesis plays a regulatory role in the heart by modulating L-type calcium channel activity via Ca2+/calmodulin-dependent protein kinase II [6]. However, additional studies are needed to expand our knowledge of the pathways that are affected by exogenously de novo synthesized FAs.

### 2.3. Reprogramming of Cardiac Metabolism in SCD1-Deficient Mice

**Figure 1:**
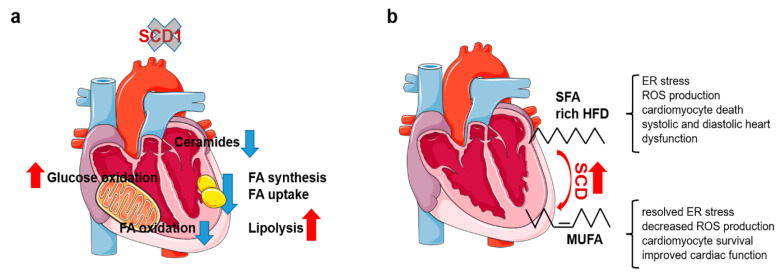
Role of SCD in the regulation of cardiac physiology and metabolism. (**a**) SCD1 deficiency stimulates a switch in substrate utilization in the heart from FA β-oxidation to glucose oxidation. Such a change may be involved in the improvement in heart function in obese leptin-deficient ob/ob mice. Additionally, SCD1 deficiency reduces de novo FA synthesis and uptake, thereby reducing the synthesis of toxic ceramides. The loss of SCD1 also reduces cardiac steatosis in PPARα-deficient mice by stimulating ATGL and HSL. (**b**) An increase in SCD activity in the heart protects against metabolic changes that are induced by a HFD with high SFA content. SCD detoxifies SFA excretions, producing MUFA. This leads to the elimination of ER stress and promotes cardiomyocyte survival, resulting in an improvement in cardiac function. The red arrow indicates the activation of the process. The blue arrow indicates the decrease of the process/decline of the content of a given factor.

Most data on SCD function in the heart come from *Scd1* knockout mice (Figure 1a). SCD1 deficiency inhibits free FA (FFA) influx and TAG synthesis in the heart without affecting cardiac performance [32,52]. Moreover, the loss of SCD1 reprograms cardiac metabolism, inhibiting FA oxidation and stimulating glucose oxidation. Mechanistically, *Scd1* knockout reduces PUFA content, inhibits PPARα, and stimulates insulin signaling [32]. SCD activity is elevated in the heart in obese leptin-deficient mice as a result of SCD4 upregulation. *Scd1* deletion rescues the cardiac phenotype of these mice, improving cardiac systolic and diastolic function, reducing steatosis, decreasing proapoptotic ceramide synthesis, and inhibiting apoptosis. Reductions in FA synthesis and transport, combined with a lower rate of β-oxidation, improved cardiac function in leptin-deficient mice [52]. The loss of SCD1 also reduces FFA, DAG, and ceramide content in *PPARα* knockout mice. Moreover, SCD1 inhibition reduces neutral lipid accumulation in stearic acid overloaded HL1 cardiomyocytes with PPARα inhibition. Thus, the antisteatotic effect of genetic/pharmacological SCD1 downregulation occurs independently of the action of PPARα but is mediated by the inhibition of SREBP-1-regulated de novo lipogenesis and by the activation of adipose tissue triglyceride lipase (ATGL) and hormone-sensitive lipase (HSL)-dependent lipolysis [51]. However, the induction of hypothyroidism in cardiomyocytes with SCD1 downregulation in vivo or in vitro leads to a greater magnitude of lipid accumulation than in control healthy cardiomyocytes. This was related to the strong activation of SREBP-1-mediated lipogenesis, with a slight increase in ATGL and HSL-mediated lipolysis and PPARα/AMPK-driven β-oxidation in cardiomyocytes with downregulated SCD1, leading to cardiac TAG accumulation [2]. Thus, SCD1 inhibition, depending on the cellular context, inhibits or activates cardiac lipogenesis in an interplay with other signaling cascades. SCD also regulates cardiomyocyte metabolism after myocardial infarction. *Scd1* and *Scd4* knockout inhibits AMPK activation in cardiomyocytes. SCD inhibition in HL1 cardiomyocytes also blunts AMPK activation and prevents lipid accumulation under hypoxic and lipotoxic conditions [53]. Therefore, the partial inhibition of SCD1 activity in humans may be a promising therapy for cardiac pathologies that are associated with excessive lipid accumulation.

### 2.4. Elevated SCD Activity Protects against Cardiotoxic Effects of SFA

An increase in SCD activity is protective against the cardiac dysfunction that is caused by an SFA-enriched HFD (Figure 1b). A HFD with high SFA content causes much more severe cardiac dysfunction than a HFD with high MUFA content. An increase in SFA content in phospholipids and a decrease in *Scd1* expression are responsible for the observed cardiac phenotype, which provokes an unfolded protein response that is attributable to ER stress [54]. SCD1 deficiency exacerbates palmitate-induced ER stress, further increasing the desaturation of membrane phospholipids and enhancing inositol-requiring enzyme 1-induced cardiomyocyte death in vitro [55]. Sirt1 deficiency predisposes the heart to diastolic dysfunction after an SFA-supplemented HFD, leading to high phospholipid unsaturation. In addition to ER stress, the expression of LXR target genes, including *Scd1*, was downregulated in hearts that lacked Sirt1. *Scd1* overexpression rescues membrane phospholipid desaturation and alleviates ER stress in cardiomyocytes with *Sirt1* deletion [34]. *Scd1* overexpression in primary neonatal cardiomyocytes suppresses palmitate-induced apoptosis. It stimulates TAGs synthesis, detoxifies SFA, and switches mitochondrial metabolism from FA oxidation, which generates high ROS levels, to glucose oxidation, which generates less ROS [35]. Interestingly, both *Scd1* overexpression and knockout stimulate glucose oxidation and inhibit FA oxidation in cardiomyocytes [32,35]. In the case of overexpression, the authors analyzed primary cardiomyocytes after acute overexpression, and the effect appears to be mediated by the downregulation of AMPK [35]. *Scd1* knockout may result in an age-dependent AMPK-independent compensatory response because the authors analyzed adult mice [32]. In contrast, the cardiac-specific overexpression of *Scd1* causes severe heart failure in mice. *Scd1* overexpression stimulates *de novo* lipogenesis via the upregulation of FAS and leads to cardiac steatosis with greater SFA accumulation. Additionally, *Scd1* overexpression causes upregulation of the angiotensin II receptor, AT1, which may be an additional pathogenic factor [39].

In conclusion, SCD activity increases during the progression from cardiac hypertrophy to heart failure and appears to be a protective mechanism to detoxify SFA. However, SCD1 deficiency preserves cardiac function in obese leptin-deficient mice by reducing the excessive rate of β-oxidation. Modulation of SCD activity may be an attractive approach for treating cardiometabolic disorders. However, to achieve beneficial effects on cardiac metabolism, SCD inhibition/activation should be strictly controlled according to the type of cardiac pathology, and the timing of this modulation needs to be determined in subsequent studies.

## 3. SCD in Vessel (patho)Physiology

Atherosclerosis is the most common multifactorial arterial pathology that can lead to the development of coronary artery disease. Obesity, HFD, and diabetes are factors that promote the development of atherosclerosis. Metabolic remodeling of smooth muscle and endothelial cells also contributes to atherosclerosis development. SCD1 regulates systemic metabolism and detoxifies SFAs. Thus, impaired SCD activity is involved in the development of atherosclerosis by modifying blood lipid composition and affecting the metabolism and signaling pathways of cells that form vessels.

### 3.1. Regulation of SCD1 Expression in Vessels

*Scd* expression is comprehensively regulated in the vasculature and has cell type-specific regulation. Such regulation allows cells to adjust their response to various factors and maintain vascular function under different conditions. Oleic acid downregulates *SCD1* expression and Δ-9 desaturation in human aortic smooth muscle (HASM) cells, but the trans isomer, elaidic acid, upregulates *SCD1* expression and activity in HASM cells and human umbilical vein endothelial cells [56,57]. α-Linolenic acid activates the farnesoid X receptor/small heterodimer partner axis, which inhibits the LXR/SREBP-1 pathway and, in turn, leads to a decrease in *Scd1* expression in macrophage-derived foam cells [58]. Palmitoleic acid induced SCD1 protein expression in human coronary artery smooth muscle cells but not in human arterial endothelial cells [59]. Shear stress stimulates *SCD1* expression in a PPARγ-dependent manner in human umbilical vein endothelial cells and in mice [60]. This upregulation of SCD protects cells from shear stress-induced apoptosis [61,62]. High inorganic phosphate and calcitriol downregulate *SCD1* expression in human aortic vascular smooth muscle cells. Maternal malnutrition during pregnancy affects microRNA expression in offspring aortas, which can lead to vascular disorders. In particular, maternal malnutrition upregulates microRNA200b, which targets and downregulates *Scd1* mRNA in aortas in 12-month-old offspring [63].

### 3.2. The Role of SCD1 in the Pathogenesis of Atherosclerosis

**Figure 2:**
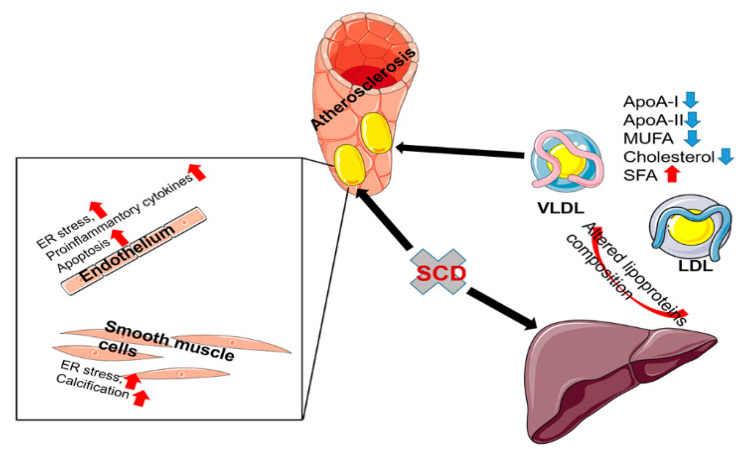
SCD1 deficiency promotes the development of atherosclerosis. SCD1 ablation modifies the composition of hepatic very-low-density lipoprotein (VLDL) and low-density lipoprotein (LDL), decreases the content of ApoA-I, ApoA-II, MUFA, and cholesterol, and increases the content of SFA, which contributes to atherosclerosis development. SCD1 ablation stimulates smooth muscle cell calcification by inducing ER stress. The inhibition of SCD1 activity in endothelial cells stimulates the secretion of proinflammatory cytokines and induces apoptosis. Altogether, this leads to the development of atherosclerosis. The red arrow indicates the activation of the process. The blue arrow indicates the decrease of the process/decline of the content of a given factor.

SCD1-deficient mice with a hyperlipidemic genetic background (i.e., low-density lipoprotein receptor knockout) that are subjected to a Western diet despite lower plasma TAG and cholesterol concentrations exhibit more severe atherosclerotic changes in the aorta (Figure 2) [64]. Moreover, a MUFA-rich HFD does not prevent the pro-atherosclerotic effect of SCD1 ablation [64]. *Scd1* silencing decreases cholesterol levels in very-low-density and high-density lipoproteins, decreases MUFA, and increases SFA levels in cholesteryl esters in these lipoproteins [65]. SCD1 ablation modifies the protein composition of high-density lipoproteins, reducing the amount of ApoA-I and ApoA-II and increasing the amount of serum amyloid A, which contributes to the development of atherosclerosis [64]. Additionally, SCD1 deficiency increased SFA content in macrophage lipids. This exacerbates the effects of proinflammatory cytokines (e.g., interleukin-1β [IL-1β] and IL-6, among others) driven by Toll-like receptor 4, contributing to the development of atherosclerosis [65]. The minor C/C genotype of the rs41290540 single-nucleotide polymorphism (SNP) of the *SCD1* gene is associated with lower total serum cholesterol levels and a lower risk of coronary heart disease in humans. This genotype in the 3′-untranslated region reduced the binding of miR-498 to *SCD1* mRNA and could lead to higher levels of its translation [66]. Remarkably, SCD inhibition has a protective effect on atherosclerosis that is induced by chronic intermittent hypoxia. The systemic inhibition of SCD1 by the administration of antisense oligonucleotides protects the aorta from atherosclerotic lesions in mice that are fed a high-cholesterol diet. The silencing of *Scd1* abolishes the increase in ultra-low-density lipoprotein C cholesterol, indicating that the protective effect of SCD1 inhibition is primarily attributable to the modification of hepatic lipid metabolism. The severity of obstructive sleep apnea positively correlates with hepatic *SCD1* expression in humans [67]. Thus, SCD inhibition may be protective or detrimental, depending on the mechanism of atherosclerosis development.

### 3.3. The Role of SCD1 in Vascular Smooth Muscle Cells Transdifferentiation

The mineralization and transdifferentiation of vascular smooth muscle cells (VSMCs) into osteoblasts are hallmarks of atherosclerosis. Exogenous or endogenous stearic acid promotes calcification and osteoblastic differentiation in the mouse aortic smooth muscle cell line (MOVAS-1). Stearate induces ER stress and activates the protein kinase R-like/eukaryotic translation initiation factor 2A/activating transcription factor 4 (ATF4) pathway, which drives osteogenic gene expression [68]. The simultaneous knockdown of *Scd1* and *Scd2* is sufficient to induce calcification in MOVAS-1 cells. Mice with the smooth muscle cell-specific dual knockout of *Scd1* and *Scd2* are characterized by vascular calcification. From a mechanistic point of view, SFA incorporates favorably with phosphatidic acid. Phosphatidic acid with fully unsaturated FA induces ER stress and, in an ATF4-dependent manner, stimulates smooth muscle cell calcification [69]. An additional mechanism of VSMC transdifferentiation into osteoblasts that is induced by SFA has been described. Palmitic acid induces the transdifferentiation of human aortic smooth muscle cells into osteoblasts via long-chain acyl-CoA synthase 3 and a nuclear factor κB (NF-κB)-dependent mechanism [70]. The knockout of elongase 6 leads to the downregulation of SCD1 in VSMCs. A decrease in SCD activity increases SFA content in cellular lipids. Elevated levels of palmitate inhibit HASM proliferation and cause their dedifferentiation via the reactive oxygen species-AMPK-Klf4 axis [71].

### 3.4. The Role of SCD1 in Endothelial Cells

Oleic acid protects human aortic endothelial cells from stearate-induced apoptosis and activation of the NF-κB inflammatory phenotype. One potent protective mechanism is oleic acid-stimulated TAG synthesis, which detoxifies stearic acid [72]. Palmitic acid is unable to induce *SCD1* expression in human arterial endothelial cells, unlike in other cell types. This makes them extremely sensitive to palmitate-induced apoptosis. The pharmacological or genetic activation of *SCD1* via an LXR receptor agonist or overexpression protects the endothelium from the lipotoxic effects of palmitate. Furthermore, SCD1 activation reduces the expression of the proinflammatory cytokines IL-6 and IL-8 under conditions of palmitate overload [59]. Palmitic acid further accentuates the tumor necrosis factor α-induced inflammatory response in the endothelial EAHy926 cell line, increasing the expression of IL-6 and IL-8. However, palmitoleic acid suppresses this response by inhibiting the expression of NF-κB target genes and activating PPARα-dependent transcription [62]. Additionally, *Scd1* overexpression increases the endothelial differentiation of bone marrow mesenchymal stem cells [73]. SCD is required for angiogenesis in the heart after myocardial infarction. The ablation of *Scd1* or *Scd4* reduces the levels of hypoxia-inducible factor 1α and vascular endothelial growth factor A in the heart after myocardial infraction. Plasma levels of proangiogenic proteins are also reduced in knockout mice. All of this leads to the inhibition of angiogenesis and may lead to poorer recovery after myocardial infarction [53]. Thus, SCD activation in VSMCs and endothelial cells appears to have a protective effect through SFA detoxification. It maintains SCD1 activity to maintain the balance between SFA and MUFA and is required for normal vascular function.

### 3.5. The Role of SCD1 in Macrophages

Cholesterol efflux from macrophage-derived foam cells is one of the protective mechanisms against atherosclerotic plaque formation. SCD inhibition stimulates cholesterol excretion from cells. Mechanistically, SCD inhibition reduces the cellular content of MUFAs, which are preferentially conjugated to free cholesterol, leading to the efflux of free cholesterol from cells [58]. Additionally, SCD-derived palmitoleic and oleic acids inhibit cholesterol efflux from J774 macrophages by downregulating ATP-binding cassette transporter A1 (ABCA1) and inhibiting its activity [74]. MUFAs increase membrane fluidity by decreasing the number of ordered regions, which can lead to a decrease in ABCA1 stability [75]. One mechanism of the antiatherogenic effect of statins occurs through the downregulation of *Scd1* expression and a decrease in MUFA content in THP-1 macrophages [76].

Thus, the action of SCD in maintaining vascular homeostasis is complex. Systemic SCD inhibition, despite its anti-obesity effect under HFD conditions, predisposes to atherosclerosis, but under certain conditions it may have an anti-atherosclerotic effect. Additionally, SCD protects VSMCs and endothelial cells from the lipotoxic effects of SFA, maintaining the smooth muscle cell phenotype and reducing the inflammatory response. Nevertheless, SCD activity inhibits the cholesterol efflux from macrophages, modifying the physical properties of the membranes.

## 4. Δ-9 Desaturation of Blood Fatty Acid as a Diagnostic Marker for Cardiovascular Disorders

### 4.1. Indirect Indicators of SCD Activity

The direct measurement of SCD activity in humans is difficult, so surrogate markers are used to assess tissue SCD activity. The product-to-substrate ratios of palmitoleic to palmitic acids (16:1/16:0) and oleic to stearic acids (18:1/18:0) in blood lipids or erythrocyte membranes are used to estimate systemic SCD activity.

An increase in blood SCD activity positively correlates with plasma TAG, obesity, and insulin resistance [77,78,79,80]. Δ-9 desaturation indices also reflect the interaction between diet and tissue SCD activity. The 16:1/16:0 ratio is higher in serum cholesteryl esters in patients on SFA-enriched HFDs compared with patients whose diet is enriched in unsaturated FA. A higher 16:1/16:0 index in patients on an SFA-rich diet is indicative of SCD activation [81]. However, dietary MUFA and PUFA reduce SCD activity as assessed by the 16:1/16:0 and 18:1/18:0 indices [82,83]. The genetic background contributes to SCD activity and is reflected in plasma FA desaturation. Several SNPs that affect plasma SCD activity have been identified. Minor genotypes for rs508834, rs10883463, rs2167444, and rs7849 SNPs decrease the 18:1/18:0 index, whereas major genotypes decrease the 16:1/16:0 FA index of serum phospholipids. The index-specific effects of these SNPs suggest that they may guide substrate selection by SCD [84]. The rs603424 SNP of *Polycystin 2 Like 1* is associated with a lower 16:1/16:0 index in phospholipid FA in erythrocytes, indicating that this gene is involved in lipid metabolism [85]. Additionally, lifestyle modifies SCD activity. Alcohol and smoking are positively associated with 16:1/16:0 and 18:1/18:0 indices, whereas physical activity and coffee consumption are negatively associated with SCD desaturation indices [83].

### 4.2. SCD Systemic Activity and Cardiovascular Health

MUFA levels, which are products of SCD, are positively associated with the development of cardiovascular disorders [86]. In a prospective population-based study, elevated levels of palmitic acid, palmitoleic acid, and oleic acid in serum ceramide esters were identified as risk factors for all-cause and cardiovascular mortality [87]. Palmitoleic acid levels are low in the diet and may be a more precise indicator of SCD activity [88]. In the Genetics of Coronary Artery Disease in Alaska Natives (GOCADAN) study, palmitoleic acid levels in red blood cell phospholipids were positively associated with an increase in heart rate, raising the heart rate by 3 beats/min for every 1% higher FA level [89]. Higher fasting serum levels of palmitic acid, palmitoleic acid, and oleic acid were found to be associated with hypertension in a Chinese post-polygon study [90]. Higher levels of palmitic acid, stearic acid, and oleic acid in ceramide esters over the long term are predictive markers of left ventricular (LV) hypertrophy in men. Higher levels of oleic acid in ceramide esters are observed in patients with both concentric and eccentric LV hypertrophy, and higher levels of palmitic acids are found in patients with concentric LV hypertrophy [91]. Additionally, elevated serum levels of palmitic, palmitoleic, and oleic acids positively correlate with the risk of stroke in middle-aged men [92]. Higher plasma levels of palmitoleic acid in cholesteryl esters and phospholipids increase the risk of heart failure in both men and women, as found in a 14-year prospective study [93].

Δ-9 desaturation ratios are better related to systemic SCD activity than levels of individual FAs. However, data on SCD activity and cardiovascular disease in humans are limited, and additional studies are needed. Metabolically unhealthy patients with a higher risk of cardiovascular disease have higher 16:1/16:0 and 18:1/18:0 ratios in whole blood samples, suggesting SCD activation. Importantly, these indices are unaffected by body mass index [94]. In the GOCADAN study, the plasma 16:1/16:0 index was positively associated with the heart rate (Table 1), but the erythrocyte 16:1/16:0 index was negatively associated with the heart rate in both men and women [95]. Additionally, SCD desaturation indicators in erythrocyte membrane phospholipid FAs are associated with elevated systolic and diastolic blood pressure in the Korean population [77]. The 16:1/16:0 ratio also increases the risk of all-cause and cardiovascular death, as found in a 20-year prospective study, and this prediction is independent of body mass index and hypertension [87]. In a 7-year follow-up study, the 18:1/18:0 index was associated with the development of heart failure, independent of diabetes or hypertension [96]. An elevated 16:1/16:0 ratio increases the risk for developing heart failure [93]. Additionally, the 18:1/18:0 index in serum phospholipids positively correlates with arterial stiffness as measured by brachial-ankle pulse wave velocity [80]. The 16:1/16:0 index is also elevated in patients with hypertension [90]. In patients with heart failure and sleep apnea, a nocturnal decrease in blood oxygen saturation is associated with a rapid increase in serum FFA [97]. Chronic intermittent hypoxia increased hepatic SCD activity in humans and mice. This leads to increases in the 16:1/16:0 and 18:1/18:0 ratios and an increase in plasma cholesterol esters and is associated with atherosclerosis [67]. There is also a strong positive correlation between the magnitude of nocturnal hemoglobin desaturation and hepatic SCD1 expression [67].

Thus, the available clinical data suggest that SCD is involved in the development and progression of cardiovascular disease. However, additional, particularly prospective studies are needed to link SCD to specific diseases for the possible development of therapeutic interventions.

## 5. Conclusions

SCD plays an important role in maintaining metabolic homeostasis of the cardiovascular system. A partial decrease in SCD activity that is related to Scd1 deficiency is cardioprotective against HFD-induced cardiac dysfunction and in leptin-deficient mice through the activation of glucose oxidation. However, SCD protects against SFA-induced cardiotoxicity by converting SFA to MUFA, thereby reducing the ER stress-induced death of cardiomyocytes and vascular endothelial cells. Nevertheless, SCD deficiency leads to atherosclerosis under hyperlipidemic conditions, accelerating vascular calcification. In humans, elevated markers of systemic SCD activity are positively associated with cardiovascular disease risk and cardiac mortality. The modulation of SCD activity appears to be an attractive therapeutic strategy. Future studies need to determine specific pathologies in which the inhibition or activation of SCD activity is applicable. Moreover, the timing of these interventions should be precisely determined because it appears that SCD activity is unchanged at the compensatory stage but increases at the decompensation stage.

## Figures and Tables

**Table 1:** Association of systemic SCD activity with cardiovascular phenotype.

Population	Parameter of Systemic SCD Activity	Association with Cardiovascular Phenotype	Reference
Middle-aged indigenous Alaskans of both sexes	Plasma 16:1/16:0	Positive association with heart rate	[95]
Middle-aged indigenous Alaskans of both sexes	Erythrocytes 16:1/16:0	Negative association with heart rate	[95]
Middle-aged Koreans of both sexes	Erythrocytes 18:1/18:0	Positive correlation with systolic and diastolic blood pressure	[77]
Men born between 1920 and 1924 and examined at 50, 60, 70, 77, and 82 years	Serum cholesterol esters 16:1/16:0	Increased hazard ratios of total and cardiovascular mortality	[87]
Seven-year follow-up study of middle-and elderly-aged subjects of both sexes	18:1/18:0	Increased hazard ratios of heart failure	[93]
Prospective study of middle-aged white subjects of both sexes from Minnesota, USA	Plasma cholesterol esters 16:1/16:0	Increased hazard ratios of heart failure	[80]
Middle-and elderly-aged Korean men	Serum phospholipids 18:1/18:0	Positive correlation with arterial stiffness	[90]
Middle-and elderly-aged Chinese of both sexes	Total serum lipids 16:1/16:0	Increased odd ratio of hypertension	[97]

## Data Availability

Not applicable.
